# Research-creation in the health sciences: A scoping review protocol

**DOI:** 10.1371/journal.pone.0359410

**Published:** 2026-10-21

**Authors:** CJ Ha, Nathan Viktor Fawaz, Liz Dennett, Susan Armijo-Olivo, Tim Barlott, Danielle Peers

**Affiliations:** 1 Rehabilitation Sciences Program, Faculty of Rehabilitation Medicine, University of Alberta, Edmonton, Canada; 2 Axiology Clinic, Edmonton, Canada; 3 Faculty of Kinesiology, Sport and Recreation, University of Alberta, Edmonton, Canada; 4 Geoffrey and Robyn Sperber Health Sciences Library, University of Alberta, Edmonton, Canada; 5 JBI University of Alberta Centre for Knowledge Mobilization, University of Alberta, Edmonton, Canada; 6 Faculty of Business and Social Sciences, University of Applied Science, Osnabrück, Germany; 7 Department of Occupational Therapy, Faculty of Rehabilitation Medicine, University of Alberta, Edmonton, Canada; Lingnan Normal University, CHINA

## Abstract

**Background:**

Research-creation is a methodological orientation that integrates creative and artistic processes with scholarly inquiry, positioning creative practice as a primary mode of knowledge generation. Originating largely within the arts and humanities in Canada, research-creation has increasingly appeared in health sciences contexts, including rehabilitation, mental health, health professions education, and community health research. However, no review has systematically mapped how research-creation is defined, conceptualized, or applied within health sciences scholarship, and significant ambiguity persists regarding the forms of data it generates, how those data are analyzed, and how creative outputs function within empirical research processes.

**Objectives:**

This scoping review aims to map how research-creation is defined and conceptualized in health sciences literature; identify the purposes for which it is used and the health-related contexts in which it appears; describe the theories, methodological approaches, and creative methods employed; and examine what forms of health-related data are generated, how they are analyzed, and how knowledge is mobilized.

**Eligibility criteria:**

Sources were included if they explicitly used the term “research-creation” (or its hyphenated and slash variants) to describe their method, approach, or project; focused on health, illness, care, healthcare systems, rehabilitation, mental health, public or community health, or health-related training; and generated empirical or experiential material analyzed or discussed in relation to health. No language limits were applied.

**Sources of evidence:**

A systematic search of 22 article databases was conducted from inception to Aug 2026. Grey literature searches included EBSCO Open Dissertations, and ProQuest Dissertations and Theses. Reference lists of included studies will be hand-searched.

**Charting methods:**

Data will be extracted independently by two reviewers using four structured charting tables capturing study characteristics, population, concept, and context. An expanded extraction framework was developed to accommodate the distinctive methodological features of research-creation, including creative processes, citational lineages, creative outputs, and forms of health-related data generated. Data will be analyzed using inductive qualitative content analysis. Extraction tools will be piloted on a subset of five included studies.

**Results:**

The search identified 842 sources, with 577 remaining after duplicate removal. Title and abstract screening, full-text screening, and data extraction are currently underway.

**Conclusions:**

This scoping review will provide the first systematic mapping of research-creation within health sciences research and training. Findings may strengthen interdisciplinary dialogue between arts, social sciences, and health disciplines; support methodological transparency for researchers employing creative inquiry; and serve as a resource for researchers, trainees, practitioners, and supervisors seeking to understand how research-creation contributes to health-related knowledge production.

## Background

Research-creation has emerged over the late twentieth century and early twenty first century as a methodological orientation that integrates creative, artistic, or practice-based processes with scholarly inquiry [1]. Originating largely within the arts, humanities, and interdisciplinary fine arts research environments in Canada, research-creation challenges traditional distinctions between knowledge generation and creative practice [2]. While definitions vary across disciplines, research-creation is generally described as an approach that combines artistic or creative activities with systematic research processes in ways that give equal weight to making, thinking, and theorizing [1,3].

Although some scholars consider research-creation and research-creative methodologies to fall within the broader category of Arts-Based Research (ABR) [4], many research-creative theorists and methodologists distinguish between research-creation and ABR through methodologically-accepted ways of considering the role of arts-based outputs associated with the research process. In ABR, arts-based methods and outputs tend to be treated as a means to particular methodological ends: secondary representations of research findings, an intervention that may be studied, something that may be engaged with at the participant level, or an important data collection mechanism in service to generating research findings. Research-creation approaches, however, position creative practice itself as a primary mode of inquiry capable of generating unique forms of knowledge [3]. Chapman and Sawchuk’s typology makes this contrast concrete, distinguishing research-for-creation, research-from-creation, creative presentations of research, and creation-as-research; only the last treats making itself as research [1]. Notably, this assertion has been institutionalized at the Canadian national research funding level through the creation of the research-creation funding stream in the early 2000s at the Social Sciences and Humanities Research Council (SSHRC) [5].

In recent years, research-creation has begun to appear within health science contexts. This emergence parallels broader methodological shifts in health research, including increased recognition of arts-based, participatory, practice-based, and multimodal forms of data and knowledge generation [6]. Health science researchers have used creative methods such as: film [7], dance [8], drama [9], participatory arts activities [10], and multimodal storytelling [11] to investigate experiences of illness, care, embodiment, disability, identity and community health. These approaches have also been applied in health science research training environments, clinical education, public health knowledge translation, rehabilitation research, health data visualization, and mental health practices [12]. Further, the therapeutic application of creative practices, such as creative art therapies, is already well-established in rehabilitation sciences and occupational therapy contexts [13].

The specific term ‘research-creation’, by contrast, appears more sparsely and across a more scattered set of fields: adapted physical activity [14], health humanities [15], public health [16], education [17], health promotion [18], health professions education [19], experiences of or within ‘ill’ or ‘sick’ bodies [20], critical data studies [21], as well as a range of critical scholarship such as disability studies and mad studies that engage and critique what is produced and socially enacted through health science research and training environments [22]. However, despite this growing presence, the concept of research-creation appears to remain undefined or underdefined and inconsistently applied in health-related literature. It is unclear how health-science researchers understand the term, how they adapt it from its arts and humanities origins, and how creative processes that are held as primary modes of inquiry function within empirical, clinical, or community-based health research and health-science research training contexts.

Research-creation may offer unique strengths to health sciences inquiry. The existing literature on ABR suggests creative processes support forms of data that are often elided in the capacities of conventional qualitative or quantitative approaches, particularly when exploring complex, affective, embodied, or culturally situated aspects of health and care [3]. Research-creation may generate experiential, sensory, or material forms of data that broaden how health-related knowledge is understood, produced, translated, and mobilized.

Reviews of ABR methods in health research exist, mapping arts-based-methods across health research [23] and their use in patient engagement [24]; neither addresses research-creation as a distinct methodological orientation. We searched PROSPERO, Google Scholar, and the University of Alberta library catalogue for prior reviews of research-creation on September 10^th^, 2026. The only review we identified addresses responsible conduct in research-creation rather than its methodology or health applications [25]. No review has systematically mapped how research-creation is defined, conceptualized, or applied within health sciences scholarship. To address this gap in literature, a scoping review is proposed to bring conceptual clarity to this emerging body of work. This review will map how research-creation is employed in health sciences research; the health-related contexts in which research-creation appears in the literature; and the stated purposes, outcomes, and forms of data associated with this work.

## Objective

The objective of this scoping review is to map how research-creation is defined, applied, and reported in health sciences research and training literature, and to characterise the forms of health-related data it generates, how those data are analyzed, and how the resulting knowledge is mobilized.

Specifically, this review will: 1) characterise how research-creation is defined and conceptualised in health sciences literature, and construct a descriptive typology of the definitions in use; 2) catalogue the stated purposes of research-creation in health sciences research and training, and map the health-related contexts, settings, and populations in which it appears; 3) describe the theoretical lineages, methodological approaches, and creative or artistic methods reported in studies that use research-creation; and 4) map the forms of health-related data research-creation generates, the analytic approaches applied to them, and the knowledge mobilisation strategies reported.

### Research questions

Our research questions are: 1) How is research-creation defined and conceptualized in health sciences literature? 2) For what purposes is research-creation used in health sciences research and training, and in which health-related contexts does it appear? 3) What theories, methodological approaches, and creative or artistic methods are described in studies that use research-creation? 4) What forms of health-related data are generated through research-creation, how are these data analyzed, and how is this knowledge mobilized?

## Methods

### Study design

This protocol describes a scoping review currently underway. The systematic search for records has been completed. The screening of records will be completed by November 2026. Data extraction will be completed by April 2027. Analysis and results will be completed by June 2027. The protocol is registered on Open Science Framework (doi:10.17605/OSF.IO/M7XYK). It was developed using the Preferred Reporting Items for Systematic Review and Meta-Analysis-Scoping review extension (PRISMA-ScR) [26] and the Joanna Briggs Institute (JBI) Scoping Review methodological framework to ensure transparency and rigour [27]. This framework consists of nine steps, the first four developed during the protocol: 1) defining the objectives and questions; 2) developing the inclusion criteria to align with the objectives and questions; 3) describing the planned approach; 4) searching for the evidence; 5) selecting the evidence; 6) extracting the evidence; 7) analysing the results; 8) presenting the results; 9) summarizing the evidence, drawing conclusions and noting any implications of the findings [27].

### Search terms and search strategies

The search strategy was developed iteratively with an experienced health sciences research librarian. Two rounds of preliminary searches and refinement were conducted by two members of the research team. Preliminary searches were conducted in September and November 2025. The first preliminary search informed how ‘health sciences’ and ‘health data’ were defined for the scoping review inclusion criteria. The second preliminary search expanded the grey literature search strategy. A systematic search of MEDLINE (via Ovid), Embase (via Ovid), APA PsycInfo (via Ovid), Global health (via Ovid), CINAHL Plus with Full Text (via EBSCOhost), ERIC (via EBSCOhost), Education Research Complete (via EBSCOhost), MLA International Bibliography (via EBSCOhost), SocINDEX with Full text (via EBSCOhost), Academic Search Complete (via EBSCOhost), Scopus, as well as the following Web of Science databases: Web of Science Core Collection, Biological Abstracts, BIOSIS Citation Index, BIOSIS Previews, CABI: CAB Abstracts, Current Contents Connect, KCI-Korean Journal Database, Policy Citation Index, Preprint Citation Index, SciELO Citation Index, and Zoological Record was performed from database inception until August 13, 2026.

The search strategy included two concepts:

1. research-creation
2. health sciences (This concept was only included in non-health sciences focused databases and included a broad swath of general health, specific disorders, health care practitioner, health facility and health services terms)

The search strategy was optimized for each database. See S1 Appendix for an example of the full list of search terms used. No limits were applied to language or date.

A search of the grey literature was performed by two team members. Because of the specific research-creation funding stream within SSHRC, we anticipated a large amount of master’s and doctoral theses as well as the potential for related published literature outside the traditional bounds of health sciences databases. Theses were searched separately in Proquest Dissertations and Theses Citation Index, and Open Dissertations using the key words “research-creation”. References of included studies will be hand searched.

We contained our search to the term “research-creation” only, rather than expanding into conceptually similar approaches such as a/r/tography or practice-based research. This was an intentional decision to limit scope creep, as well as to purposefully examine how the specific term “research-creation” is being used and understood. We acknowledge that this may exclude studies using comparable methodologies.

For feasibility purposes, we contained the search to title, abstract and keywords only, which may be a limitation of this study. Full-text searching rather than title, abstract and keywords might benefit the interdisciplinary nature of our topic of research-creation. Some reasons may include funding considerations (e.g., not wanting the work defined as research-creation for funding stream purposes). Further, the expansive and less-than-straight-forward nature of research-creative work may be such that “research-creation” does not necessarily show up in the title, abstract or keywords.

The search identified 842 articles. Covidence found and removed 265 duplicates. There are now 577 articles for screening.

### Selecting studies for analysis

Inclusion criteria were developed using insights from the preliminary searches. The inclusion/exclusion criteria for this scoping review are as follows:

1. Research-creation: For inclusion, the article *explicitly uses the term “research-creation”, “research creation”, or “research/creation” to describe its method, approach, or project. For pieces that only mention research-creation theoretically but without practical application, they will be tracked and labeled with the label “conceptual only”.
2. Health sciences/health-related: For inclusion, research is considered health sciences/health-related if its primary focus is on health, illness, care, healthcare systems, rehabilitation, mental health, public or community health, or health-related training of practitioners, and involves health-related participants, settings, or outcomes.
3. Health data/health-science data: We will include studies that generate any form of empirical or experiential material (e.g., interviews, observations, participatory/arts-based outputs, practice-based reflections, visual/audio artefacts treated as data) that are analyzed or discussed in relation to health, illness, care, or health-related practice/training.

### Screening for relevant articles

Two independent team members will screen title and abstract followed by full text screening. We will pilot the screening process on a subset of potentially relevant sources to determine agreement (the first 25 sources), aiming for a minimum of 80% interrater agreement amongst the screeners. In the event of disagreement, the two team members will come to an agreement through discussion. If consensus cannot be reached, a third research team member will be called in to make the final agreement. There may be opportunity at this stage to amend our eligibility criteria if needed. PRISMA-ScR will be used for selection flow and to illustrate all excluded studies and reasons. The search may be modified and expanded during the review process to account for new potentially relevant terms and concepts that may emerge. The team members are using Covidence software to organize, screen, and extract data.

### Data extraction

All included study data will be extracted using Tables 1–4 below (see S2 Appendix for data extraction guides). We will pilot our extraction tools on a subset of 5 included studies to test its feasibility as suggested by the JBI Scoping review protocol [27]. We are including the pilot according to JBI guidelines, and anticipate that due to the iterative, multimodal and self-referential nature of research-creation, we may find that our selected extraction categories need adjustment. The final completed tools will be presented in the scoping review report. Data extraction will occur independently by two team members. Disagreements will be resolved through discussion. We anticipate these discussions will be complex in nature and will require several conversations with the larger team.

**Table 1:** Data extraction: Study characteristics.

Citation	Year	Country	Funding source	Study design	Study size	Ontological/Epistemological positioning	Project iteration
							
							

**Table 2:** Data extraction: Population.

Researchers	Trainees	Participants in RC	Populations described	Communities	Patient groups	Health conditions
						
						

RC: research-creation

**Table 3:** Data extraction: Concept.

RC definition	RC purpose	RC lineage	Methodology	Creative processes	Creative outputs	Health-related data	Methods of data analysis	KM
								
								

KM: knowledge mobilization; RC: research-creation

**Table 4:** Data extraction: Context.

Health Discipline	Health Sciences Setting	Health-related Topic	Training/Educational Context	Health-related Data as Material
				
				

### Data extraction approach rationale

We have developed an expanded extraction framework to support translation between research forms because research-creation involves methodological features that differ substantially and substantively from conventional qualitative, quantitative, or mixed-methods health-science research, including: iterative cycles of making and reflection, multimodal forms of data generation, creative artifacts functioning simultaneously as process and output, multiple lineages of theoretical grounding, and the positioning of researchers as artists. These features require attention to elements not usually held in scoping review extraction tools (e.g., citational lineages, creative processes) and so our extraction categories intentionally foreground the conceptual, methodological, and creative dimensions of research-creation alongside traditional health-science descriptors (e.g., population, study design, analytic approach). We hope this structure allows us to demonstrate how research-creation functions as inquiry within health sciences settings without imposing conventional empirical molds but still enabling systematic comparison across studies.

### Data analysis

Data will be analyzed using an inductive approach for all of the research questions. Using basic qualitative content analysis for scoping reviews by Elo and Kyngäs [27], we will approach the data using the three phases described (preparation, organization, and reporting). Following this framework on data analysis, we will do open coding of the data to create categories for the purposes of identifying and clarifying definitions and concepts. To increase transparency on the coding process, a pre-coded version of the extraction table, as well as the code guide our research team has determined from the extracted data will be published to the project file folder in Open Science Framework (https://osf.io/yazck/overview).

Data visualization is a core component of knowledge mobilization both within the research community and between researchers and interested members of the public [28]. Calls for creative forms of data visualization, beyond tabular formats, have been made by members of the research community specializing in evidence synthesis [28,29]. With training and experience in the fine arts, two members of this working team are uniquely suited to develop creative visual forms for data representation. The analyzed data will be presented in various ways, including tables alongside more visually creative formats, depending on findings.

### AI disclosure

Claude (Anthropic) was used at two stages of manuscript preparation. It produced the initial draft of the abstract and the cover letter. Following peer review, it was used to compare the manuscript’s stated objectives against its research questions, to identify redundancy in the background, and to draft candidate replacement wording for those sections.

The validity of these outputs was evaluated by CJH and NVF. Each AI-drafted passage was checked against the corresponding section of the manuscript and against the reviewer comment it was intended to address. All bibliographic claims, including proposed corrections to reference details, were verified against the original published sources before being accepted. Proposed wording was revised or rejected where it did not reflect the authors’ intended meaning.

Generative AI was not used to: develop or execute the search strategy, screen records, extract data, or analyse findings; these steps were and will be carried out by two independent team members as described above. No AI tool generated or altered data or supporting files. All statements of objectives, interpretations, limitations, and implications represent the authors’ own ideas. Claude is not an author and made no authorship-level contribution. The authors are fully responsible for all content of this manuscript, including portions drafted or revised with AI assistance.

## Implications

This scoping review is the first of its kind to map research-creation and how it is being used in health sciences research and training. Implications of our findings can strengthen interdisciplinary, transdisciplinary, and transparadigmatic dialogue between arts, social sciences, fine arts, and health sciences disciplines; support methodological transparency; and provide a resource to researchers, trainees, practitioners, and supervisors to better understand how research-creation contributes to health-related knowledge production. It may also inform future methodological development, training, and evaluation of creative inquiry within health sciences research.

## Supporting information

S1 Appendix Example of search terms. Contains the full search terms used for Scopus (Elsevier).
(DOCX)

S2 Appendix Data extraction guide. Contains data extraction guidelines for all four data extraction tables.
(DOCX)

S1 File Preferred Reporting Items for Systematic Reviews and Meta-Analyses Extension for Scoping Reviews (PRISMA-ScR) checklist. Contains the PRISMA ScR checklist details.
(DOCX)

## References

[1] Chapman O, Sawchuk K. Research-creation: intervention, analysis and “family resemblances.”. Can J Commun. 2012;37(1):5–26. doi: 10.22230/cjc.2012v37n1a2489

[2] Manning E. Against method. In: Manning E, editor. The minor gesture. United States: Duke University Press; 2016. 26–45.

[3] Truman SE. Undisciplined: Research-creation and what it may offer (Traditional) qualitative research methods. Qual Inq. 2023;29(1):95–104. doi: 10.1177/10778004221098380

[4] Leavy P. Method meets art: Arts-based research practice. New York: The Guilford Press; 2020.

[5] SSHRC. Social sciences and humanities research council. 2025. Accessed 2026 January 5. https://sshrc-crsh.canada.ca/en/funding/opportunities/resources/preparing-application-involving-research-creation.aspx

[6] Hinsliff-Smith K, McGarry J, Ali P. Arts based health care research: A multidisciplinary perspective. Cham, Switzerland: Springer International Publishing; 2022.

[7] Giling R, Heerings M, Bovenkamp HM van de. Art of conveying an experience: A visual anthropological approach to understand how film can convey patient experience. Med Humanit. 2025. doi: 10.1136/medhum-2025-013555 41314795

[8] Guzmán-García A, Mukaetova-Ladinska E, James I. Introducing a Latin ballroom dance class to people with dementia living in care homes, benefits and concerns: A pilot study. Dementia (London). 2013;12(5):523–35. doi: 10.1177/1471301211429753 24337327

[9] Brown J, Carr V, Smith AC, Gardner M, Hjorngaard-Ferentzi M, Mykitiuk R. Small acts of saying. Worlding difference. 2024. https://worldingdifference.ca/video/small-acts-of-saying/

[10] Crone DM, O’Connell EE, Tyson PJ, Clark-Stone F, Opher S, James DVB. “Art Lift” intervention to improve mental well-being: An observational study from U.K. general practice. Int J Ment Health Nurs. 2013;22(3):279–86. doi: 10.1111/j.1447-0349.2012.00862.x 22897659

[11] Heerings M, van de Bovenkamp H, Cardol M, Bal R. Ask us! Adjusting experience-based codesign to be responsive to people with intellectual disabilities, serious mental illness or older persons receiving support with independent living. Health Expect. 2022;25(5):2246–54. doi: 10.1111/hex.13436 35178839 PMC9615044

[12] Wairimu HK. Innovative approaches to integrating arts in medical education. Eurasian Exp J Sci Appl Res. 2024;5(3):66–9.

[13] Levy CE, Uomoto JM, Betts DJ, Hoenig H. Creative arts therapies in rehabilitation. Arch Phys Med Rehabil. 2025;106(1):153–7. doi: 10.1016/j.apmr.2024.07.008 39043332

[14] Eales L. Loose leaf. Can J Disabil Stud. 2016;5(3):58. doi: 10.15353/cjds.v5i3.297

[15] Watson A, Wozniak-O’Connor V, Lupton D. Health information in creative translation: establishing a collaborative project of research and exhibition making. Health Sociol Rev. 2023;32(1):42–59. doi: 10.1080/14461242.2023.2171802 36759339

[16] Sadati SMH, Mitchell C. Serious game design as research-creation to address sexual and gender-based violence. Int J Qual Methods. 2021;20. doi: 10.1177/16094069211046130

[17] Martin K. Embodiment in an indigenous lit classroom: Why I’m over discussion but can’t get enough of research-creation. Stud Am Indian Lit. 2022;34(1–2):16–29. doi: 10.1353/ail.2022.0003

[18] Beck I. In(k)tervention. Health Promot Pract. 2026;27(2):217–20. doi: 10.1177/15248399251370372

[19] Brand G, Peters S, Dart J. Verbatim theatre as a creative approach to health professions education research translation. Med Educ. 2024;58(11):1296–303. doi: 10.1111/medu.15449 38922957

[20] Hedva J. How to tell when we will die: on pain, disability, and doom. New York: Zando; 2024.

[21] Lupton D, Watson A. Towards more-than-human digital data studies: Developing research-creation methods. Qual Res. 2021;21(4):463–80. doi: 10.1177/1468794120939235

[22] Foisy C. Sounding mad poetics as resistant-methods and research-creation. Disabil Stud Q. 2025;45(1). doi: 10.18061/dsq.6913

[23] Fraser KD, al Sayah F. Arts-based methods in health research: A systematic review of the literature. Arts Health. 2011;3(2):110–45. doi: 10.1080/17533015.2011.561357

[24] Phillips EK, Chudyk AM, Monnin C, Schultz ASH, Arora RC, Duhamel TA, et al. The use of arts-based methods to enhance patient engagement in health research. Health Expect. 2024;27(6):e70127. doi: 10.1111/hex.70127 39679770 PMC11647696

[25] Voarino N, Couture V, Mathieu-Chartier S, Bélisle-Pipon JC, St-Hilaire E, Williams-Jones B, et al. Mapping responsible conduct in the uncharted field of research-creation: A scoping review. Account Res. 2019;26(5):311–46. doi: 10.1080/08989621.2019.1620607 31134814

[26] Peters MDJ, Godfrey CM, Khalil H, McInerney P, Parker D, Soares CB. Guidance for conducting systematic scoping reviews. Int J Evid Based Healthc. 2015;13(3):141–6. doi: 10.1097/XEB.0000000000000050 26134548

[27] Pollock D, Evans C, Jia RM, Alexander L, Pieper D, Brandão De Moraes É, et al. “How-to”: scoping review?. J Clin Epidemiol. 2024;176:111572. doi: 10.1016/j.jclinepi.2024.111572 39426499

[28] South E, Rodgers M. Data visualisation in scoping reviews and evidence maps on health topics: A cross-sectional analysis. Syst Rev. 2023;12(1):142. doi: 10.1186/s13643-023-02309-y 37587522 PMC10433592

[29] Pollock D. A practical guide to scoping reviews: Purpose and process. 2025. https://www.youtube.com/watch?v=l5bRbo7gqt4

