# Supplementary material for: Research-creation in the health sciences: A scoping review protocol
Source: PLoS One. 2026 Oct 21;21(10):e0359410. doi: 10.1371/journal.pone.0359410 (PMC13686477; doi:10.1371/journal.pone.0359410)
Supplement: Supplementary file 1 [file pone.0359410.s001.docx]

**S1 Appendix**

Search terms for Scopus (Elsevier)

Advanced search

Date searched: Aug 13, 2026

Results 161

TITLE-ABS-KEY("research creation") AND  TITLE-ABS-KEY(**health* or healthcare or medic* or**  **clinic*** or **therap* or treatment*** **or disease* or disorder*** or **injur* or pain or disab* or illness*** **or psychiatr*** **or patient or patients** or inpatient* or prescription* or pharma* or wellness or wellbeing or well-being or quality-of-life or QoL or HRQOL* or "social determinants" or  "functional status" or "functional impairment*" or "activities of daily living" or mortalit* or death* or fatalit* or dying  or aging or  convalesc* or outbreak* or infectio*  or patholog* or pathogen* or virus*  or epidemic* or pandemic* or deformit* or frailty or birth-outcome* or fever* or symptom* or dizziness or dizzy or vertigo or faint or fainting or fatigue or syncope or drows* or "chronic condition*" or "chronic care" or "acute condition*" or "acute care" or comorb* or morbidit* or prophylaxi* or cancer* or neoplasm* or tumour* or tumor* or sarcoma* or myeloma* or leukemia* or melanoma* or lymphoma* or malignanc* or heart-attack* or "heart failure" or "myocardial infarction*" or hypertensi* or "atrial fibrillation" or stroke* or asthma* or allerg* or epilep* or anorexi*  or COPD or cirrhosis or pneumonia or tuberculosis or measles or meningitis or syphilis or typhoid or dengue or encephalitis or parasite* or parasitic or malnutrition or hemorrhag* or malaria or hepatitis or hiv or "hiv/aids" or aneurysm* or embolism* or "multiple sclerosis" or hernia* or neuropath* or "kidney stones" or arthriti* or osteoarthriti* or fibromyalgi* or "chronic fatigue" or "Myalgic Encephalomyelitis" or lupus or gout or dialysis or migraine* or headache* or obes* or diabet* or alzheimer* or parkinson* or dementia  or surger* or surgical or abortion*  or blindness or "visual impairment*" or cataracts or "hearing loss" or glaucoma or  parapleg* or quadrapleg* or crip or crips or cripple* or "hearing impairment*" or deafness or deaf or wheelchair* or autis* or "attention deficit*" or adhd or auadhd or tourette* or "down syndrome*" OR "cystic fibrosis" or "fetal alcohol" or neurodiver* or "stress level*" or "high* stress*" or "psychological stress*" or "mental stress*" or burnout  or depression or antidepressant* or "anti-depressant*" or anxiety or stress-disorder* or PTSD or trauma* or madness or mad or psychosis or psychoses or psychotic or schizophreni* or bipolar or ocd or obsessive-compulsive or phobia* or phobic or paraphili* or abuse  or insomnia* or addict* OR opioid* OR heroin OR drug OR drugs OR fentanyl OR narcotic* OR opiate* OR methadone OR heroin OR cocaine OR hallucinogen* OR marijuana OR cannabis OR nicotine OR tobacco OR psychedelic* OR psilocybin OR ( ( substance* OR stimulant* or alcohol*) W/3( "use" OR user* OR usage OR abuse* OR misuse* OR addict* OR disorder* OR dependen* OR withdrawal ) ) OR alcoholi* OR "gambling disorder*" OR "problem gambling" OR "compulsive buying" OR "compulsive shop*" OR "compulsive sexual behavior" or  hospice or "palliative care" or "end of life care" or "longterm care" or "long term care" or "access to care" or "care seeking" or "primary care" or "urgent care" or "tertiary care" or hospital* or "emergency department*" or "emergency care" or "emergency room*" or "critical care" or "intensive care" or ICU or NICU or rehab* or "home care" or telehealth or telecare or telemedicine or ehealth or mhealth or telerehab* or telepsych* or  "early detection" or "early intervention*" or "preventative care" or "primary care" or immunization* or immune or autoimmun* or immunocompromi* or immuno-compromi* or vaccin* or antivax or "self care" or "self management" or ambulance or physician* or "general practitioner*" or caregiver* or nurs* or doctor* or surgeon* or hospital* or clinic* or primary-care or medical-care or emergency-room* or doctor* or physician*  or nurs* or psycholog* or physiotherap* or anaesthe* or anesthes* or cardiolog* or dermatolog* or endocrinolog* or endoscop* or gastroenterolog* or geriatric* or gerontolog* or gynaecolog* or gynecolog* or haematolog* or hematolog* or hepatolog* or  immunolog* or internist* or neonatolog* or nephrolog* or neurolog* or neurosurgeon* or obstetric*  or oncolog* or ophthalmolog* or orthopedi* or otolaryngolog* or paediatric* or pediatric* or patholog* or podiatr or physiatr* or psychiatr* or proctolog* or pulmonolog* or radiolog* or rheumatolog* or urolog* )
