# Supplementary material for: Research-creation in the health sciences: A scoping review protocol
Source: PLoS One. 2026 Oct 21;21(10):e0359410. doi: 10.1371/journal.pone.0359410 (PMC13686477; doi:10.1371/journal.pone.0359410)
Supplement: Supplementary file 2 [file pone.0359410.s002.docx]

**S2 Appendix**

**Table 1 Guide: Study Characteristics**

| **Column** | **Description** |
| --- | --- |
| Citation | Full citation (Vancouver style). |
| Year of Publication | Numerical year. |
| Country/Region | Country where the study was conducted or contextually situated. |
| Funding Source(s) | Named funders; indicate “SSHRC RC stream” if relevant. |
| Study Design / Type | Qualitative, practice-led research, multi-iterative RC project, research-creation lab, case study, community-based RC project. |
| Ontological/Epistemological Positioning | (If stated) e.g., emic/etic, inductive/deductive, post-qualitative, materialist, etc. |
| Study Size | Number of participants or number of creator-researchers. |
| Project Iteration Structure | Note number, type, or logic of iterations (e.g., iterative prototyping, cycles of performance–reflection, emergent recursive making). |

RC: research-creation; SSHRC: Social Sciences and Humanities Research Council

Note on project iterations: Research-creation projects often involve multiple cycles of creation and reflection. Unlike participatory action research, iteration is not always linear or discrete.

**Table 2 Guide: Population**

Reflects *who* or *what* is represented within the research-creation literature. Not all categories will apply to all studies.

| **Column** | **Description** |
| --- | --- |
| Researchers | How researchers are positioned (e.g., researcher-as-artist, co-creators). |
| Trainees | Roles of students, interns, residents, etc. |
| Participants in RC projects | Demographics and characteristics of participants engaged in creative inquiry |
| Populations described | Clinical/health populations (e.g., stroke survivors, autistic youth). |
| Communities | Community partners, collectives, cultural groups. |
| Patients/Groups | Patient groups directly represented. |
| Health Conditions | If conditions are explicitly connected to the inquiry. |

RC: research-creation

Notes: Some papers may not have “participants” in a classical sense, e.g., multi-autoethnographic RC projects centered on researchers’ own practice. These should be coded distinctly (e.g., self-study, autoethnographic, process-centered).

**Table 3 Guide: Concept**

Includes all elements needed to answer RQs 1–4.

| **Column** | **Description** |
| --- | --- |
| Definition of Research-Creation | How RC is defined, cited, framed; quote key phrases. |
| Purpose of RC in the Study | What RC is for (data generation, KT, pedagogical tool, intervention, etc.). |
| RC Citational Lineage/Other Theoretical Roots | E.g., Manning & Massumi, Chapman & Sawchuk, critical disability studies, new materialisms, Indigenous methodologies. |
| Methodological Approach | Qualitative, mixed, practice-based research, sensory ethnography, participatory. |
| Creative/Artistic Methods & Processes | E.g., film, digital media, textile work, choreography, performance ethnography, sonic inquiry, etc. |
| Creative Output(s) | Artifacts, performances, films, installations, digital works, etc. |
| Forms of Health-Related Data Generated | E.g., sensory data, experiential reflections, creative artifacts treated as data, embodied practices, interviews, observational data, material traces. |
| Method of Data Analysis | How data – including creative data – are analyzed (e.g., thematic analysis, diffractive analysis, aesthetic analysis, practice-led reflection, multimodal coding). → Real question here…is data always analyzed in RC is the RC process itself a form of analysis? |
| Knowledge Mobilization | Explicit KM strategies; how creative outputs circulate. |

KM: knowledge mobilization; KT: knowledge translation; RC: research-creation

Note: *Forms of health-related data* and *analysis strategies* may not be obvious until piloting.

**Table 3 Guide: Context**

Captures how and where health-science environments interface with research-creation.

| **Column** | **Description** |
| --- | --- |
| Health Discipline / Field | E.g., nursing, OT, PT, public health, mental health, rehab, kinesiology. |
| Health Sciences Setting | Clinical, community-based, educational, lab, simulation, arts-health environment. |
| Health-Related Topic | Illness experience, care, disability, embodiment, health identity, community health. |
| Training / Educational Context | Whether and how RC is used for teaching, supervision, curriculum. |
| Health-related data as material | Utilization of health-related data in artistic practice. |

OT: occupation therapy; PT: physiotherapy; RC: research-creation; rehab: rehabilitation
